# Supplementary material for: Metabolic Syndrome but Not Fatty Liver-Associated Genetic Variants Correlates with Glomerular Renal Function Decline in Patients with Non-Alcoholic Fatty Liver Disease
Source: Biomedicines. 2022 Mar 19;10(3):720. doi: 10.3390/biomedicines10030720 (PMC8944982; doi:10.3390/biomedicines10030720)
Supplement: Supplementary file 1 [file biomedicines-10-00720-s001.zip › biomedicines-1641360-supplementary materials.pdf]

Table S1. Multivariate analyses of factors associated with reduced GFR including PNPLA3 recessive and additive model and unweighted GRS.

Panel A. Factors Associated with eGFR < 90 mL/min/1.73m2			
**Variables**	**Model A OR (95% C.I.)**	**Model B OR (95% C.I.)**	**Model C OR (95% C.I.)**
BMI	0.97 (0.93–1.00)	0.97 (0.93–1.00)	0.97 (0.93–1.00)
Metabolic Syndrome	1.56* (1.08–2.24)	1.56* (1.08–2.25)	1.58* (1.10–2.27)
FIB4−	0.85 (0.53–1.34)	0.87 (0.55–1.39)	0.87 (0.55–1.38)
PNPLA3 GG	0.73 (0.43–1.26)	-	-
PNPLA3 GG vs. CC		1.23 (0.85–1.77)	
PNPLA3 GG vs. CG		0.81 (0.46–1.44)	
Unweighted GSR	-	-	0.81 (1.02–1.17)

Model A: including BMI, metabolic syndrome, *PNPLA3* GG and FIB4 -. Model B: including BMI, metabolic syndrome, *PNPLA3* GG vs. CG and *PNPLA3* GG vs. CG and FIB4−. Model C: including BMI, metabolic syndrome, unweighted GSR and FIB4−. *$p < 0.05$

Table S2. Multivariate analysis including age as covariate.

Panel A. Factors Associated with eGFR < 90 mL/min/1.73m2					
**Variables**	**Univariable Analysis OR Ɨ(95% C.I.)**	**Model A OR (95% C.I.)**	**Model B OR (95% C.I.)**	**Model C OR (95% C.I.)**	**Model D OR (95% C.I.)**
PNPLA3 CG/GG	1.16 (0.83–1.63)	1.25 (0.87–1.81)	1.30 (0.90–1.87)	1.33 (0.92–1.93)	-
Metabolic Syndrome	1.42* (1.00–2.00)	1.11 (0.75–1.64)	-	-	1.11 (0.75–1.64)
High blood pressure#	1.48* (1.01–2.15)	-	0.94 (0.62–1.44)	-	-
Arterial hypertension	1.47* (1.04–2.08)	-	-	0.90 (0.61–1.33)	-
Weighted GSR	1.24 (0.64–2.39)	-	-	-	1.45 (0.72–2.93)
**Panel B. Factors Associated with eGFR < 60 mL/min/1.73m2**					
**Variables**	**Univariable Analysis OR (95% C.I.)**	**Model A OR (95% C.I.)**	**Model B OR (95% C.I.)**	**Model C OR (95% C.I.)**	**Model D OR (95% C.I.)**
PNPLA3 CG/GG	0.98 (0.48–2.01)	0.80 (0.53–2.26)	1.14 (0.55–2.40)	1.18 (0.57–2.45)	-
Arterial Hypertension	2.57* (1.09–6.06)	-	-	1.78 (0.72–4.39)	-
Weighted GSR	0.92 (0.23–3.65)	-	-	-	1.77 (0.45–6.95)

Model A: adjusted for age, BMI, metabolic syndrome, *PNPLA3* GG/CG and FIB4 -. Model B: adjusted for age, high blood glucose, high waist circumference, high blood pressure, low HDL cholesterol, high triglycerides, *PNPLA3* GG/CG genotype and FIB4 -. Model C: including arterial hypertension instead of high blood pressure, diabetes instead of high blood glucose, high waist circumference, low HDL cholesterol, triglycerides, age, *PNPLA3* GG/CG genotype and FIB4 -. Model D: adjusted for age, BMI, metabolic syndrome, weighted GRS and FIB4−. $^{*}p < 0.05$; # according to ATPIII modified criteria.

Table S3. Univariate correlation between age, MetS and arterial hypertension.

Variables	Metabolic Syndrome OR (95% CI)	Arterial hypertension OR (95% C.I.)
Age	1.04 (1.02–1.05)°	1.07 (1.05–1.09)°

°$p < 0.001$.

Table S4. Propensity scores match of patient with *PNPLA3* GG and CC genotypes according to age, sex, metabolic syndrome and arterial hypertension.

Variables	*PNPLA3* GG (n = 64)	*PNPLA3* CC (n = 64)	p
Age (years)	53.0 ± 11.7	53.5 ± 10.6	0.816
Female (%)	43.8	40.6	0.720
Metabolic Syndrome (%)	46.9	46.9	1.000
Arterial Hypertension (%)	48.4	46.9	0.860
eGFR (mL/min/1.73m^2)	93.7 (82.0–104.8)	94.6 (81.2–104.2)	1.000

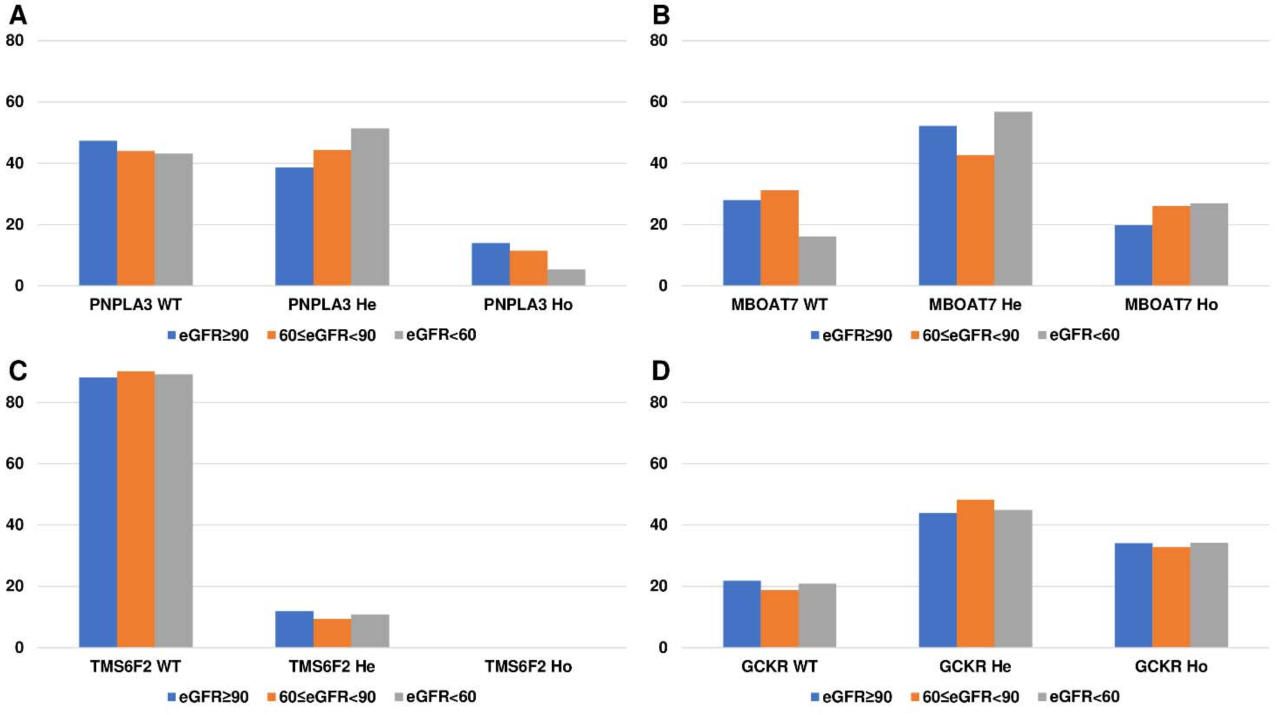

Figure S1. Genotypes prevalence according to eGFR. Panel (**A**) shows prevalence of *PNPLA3* wild type (WT), heterozygous (He) and homozygous (Ho) according to eGFR cut-offs; Panel (**B**) shows prevalence of *MBOAT7* Wt, Hz and Ho according to eGFR cut-offs; Panel (**C**) shows prevalence of *TM6SF2* Wt, Hz and Ho according to eGFR cut-offs; Panel (**D**) shows prevalence of *GCKR* Wt, Hz and Ho according to eGFR cut-offs.

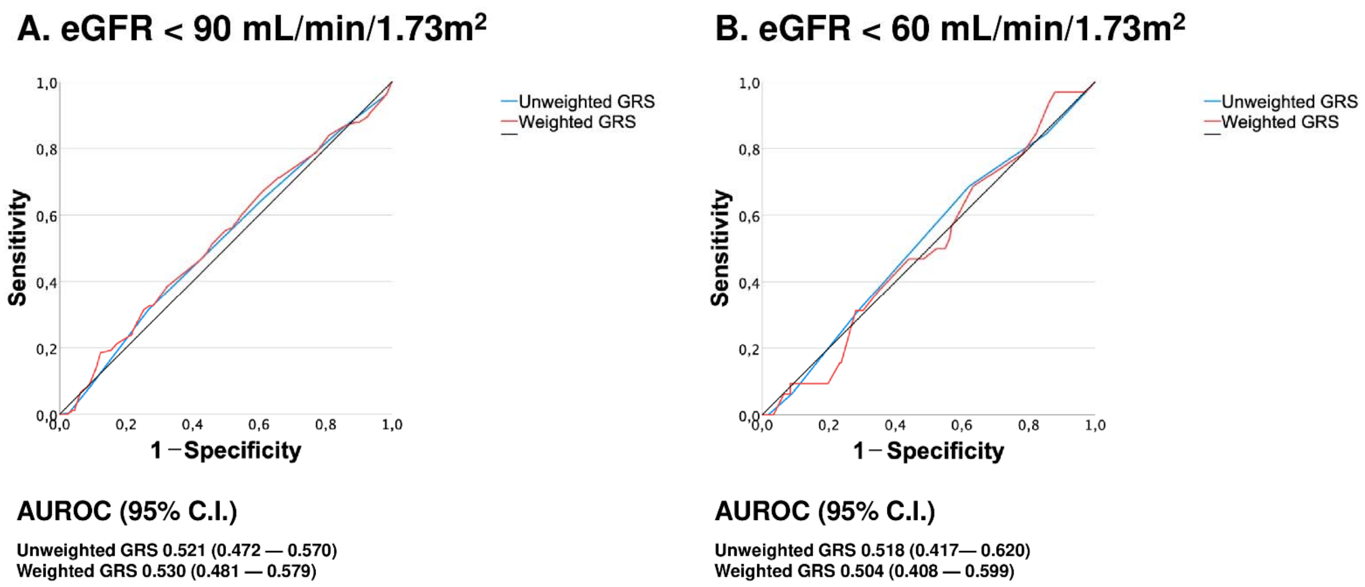

Figure S2. ROC curve of unweighted and unweighted GRS for the detection of GFR <90 mL/min/1.73m^2 (**A**) and of GFR < 60 mL/min/1.73m^2 (**B**).
